# CNS-compartmentalized IgG aggregates and glycosylation in multiple sclerosis contribute to oligoclonal bands and neuronal cytotoxicity

**DOI:** 10.3389/fimmu.2026.1689835

**Published:** 2026-03-20

**Authors:** Sakthi Asokan, Wenbo Zhou, Anthony Fringuello, Tiffany Pointon, Ashley Kennedy, Brendan Freitas, Jackson Tumas French, Haiyan Zhao, Christina Coughlan, Enrique Alvarez, Xiaoli Yu

**Affiliations:** 1 Department of Neurosurgery, University of Colorado Anschutz Medical Campus, Aurora, CO, United States; 2 Department of Neurology, University of Colorado Anschutz Medical Campus, Aurora, CO, United States; 3 Department of Biochemistry and Molecular Genetics, University of Colorado Anschutz Medical Campus, Aurora, CO, United States

**Keywords:** autoantibodies, cerebrospinal fluid, complement activation, glycosylation, IgG aggregates, multiple sclerosis, neuroinflammation, neuronal cytotoxicity

## Abstract

**Background:**

Oligoclonal bands (OCBs) are a hallmark of multiple sclerosis (MS), yet their molecular characteristics and pathogenic relevance remain incompletely understood. Recent evidence suggests that immunoglobulin G (IgG) aggregates and glycosylation may contribute to neuroinflammation and neuronal injury in MS.

**Methods:**

We analyzed paired cerebrospinal fluid (CSF) and plasma samples from MS patients and other neurological controls using transmission electron microscopy, protein aggregation assays, proteomics, isoelectric focusing immunoblotting, and Western blots. Neuronal cytotoxicity was assessed using human iPSC-derived neurons and SH-SY5Y cells. IgG glycosylation was evaluated by enzymatic deglycosylation and lectin-based detection.

**Results:**

We identified large IgG aggregates (> 100 nm) in MS CSF, which were absent in controls and induced complement-dependent neuronal apoptosis. These aggregates were enriched in OCBs and were disrupted by urea or glycine-HCl, resulting in the loss of OCBs. Proteomic analysis revealed enrichment of IgG subclasses and complement components in MS CSF. In addition, MS CSF contained significantly elevated levels of galactosylated and sialylated IgG compared to paired plasma. Enzymatic removal of glycans reduced both OCB intensity and neuronal cytotoxicity.

**Conclusions:**

Our findings demonstrate that CNS-compartmentalized IgG aggregates and glycosylation contribute to the formation of OCBs and neuronal cytotoxicity in MS. These results provide new insights into the molecular basis of OCBs and suggest that targeting IgG glycosylation or aggregation may offer novel therapeutic strategies for MS.

## Introduction

Compartmentalized inflammation has been recognized as an essential feature of multiple sclerosis (MS), where the immune system’s response becomes localized within the central nervous system (CNS) (1, 2). The compartmentalized inflammatory response in the MS brain is composed of tissue-resident CD8+ T lymphocytes and B cells (3). Microglia, the CNS-resident macrophages, play a critical role in compartmentalized inflammation and contribute to local immune activation in MS (3, 4). Oligoclonal bands (OCBs), the characteristic immunopathological features of MS (4, 5), are persistently present in MS CSF (6) and recognize identical epitopes over time (7). Increased cortical lesion load and intrathecal inflammation are associated with MS OCBs (8). CNS compartmentalized inflammation in MS affects immunoglobulin G (IgG)1 fragment crystallizable (Fc) glycosylation, showing features that enhance Fc effector functions (9).

Protein aggregates are common in neurodegenerative diseases such as Alzheimer’s disease and Parkinson’s disease (9, 10). We previously reported that, in MS, the large IgG aggregates (> 100 nm) in the blood can induce complement-dependent neuronal cytotoxicity (11). Although several lines of research have suggested the presence of IgG aggregates in the MS central nervous system (12–14), direct evidence of their presence in MS cerebrospinal fluid (CSF) has not been previously reported, and their pathological role remains to be elucidated.

IgG effector function has been increasingly appreciated for its pathological roles in autoantibody-mediated diseases. Posttranslational glycosylation of the IgG constant region contributes to the pathology of these disorders. Both *N*-linked and *O*-linked glycans are present in IgG molecules. Studies have shown that IgG isotype- and subclass-specific glycosylation occurs. Notably, EndoS, an endoglycosidase secreted by *Streptococcus pyogenes*, removes *N*-linked glycans from the heavy chain of human antibodies. EndoS has successfully treated numerous autoimmune conditions in animal models (10). IgG glycosylation in both the Fc and fragment antigen-binding (Fab) regions significantly modulates IgG activity by influencing its stability, half-life, and effector functions (11). Fab *N*-glycan composition affects IgG aggregation and precipitation (12). As an essential part of the Fc glycan, terminal galactose affects protein conformation (13), complement activation, and enhances complement-dependent cytotoxicity (14). Fc galactosylation has been shown to promote IgG1 hexamerization, leading to enhanced classical complement activation (15). In MS, the abundance of glycosylated intrathecally synthesized IgG is the most prominent trait and is associated with higher MRI lesion load (16). MS CSF IgG1 glycosylation has features that enhance Fc effector functions (9). We and others have reported alterations of IgG glycosylation in MS (17–19), supporting its proinflammatory potential.

In the current study, we demonstrate the presence of IgG aggregates in MS CSF, which induce neuronal cytotoxicity. We further show that MS CNS-compartmentalized IgG antibodies contain elevated levels of glycans and contribute to oligoclonal bands and neuronal cytotoxicity. Our data support the unique features of IgG antibodies in MS (20), which contribute to the CNS-compartmentalized inflammation and play key roles in disease pathogenesis.

## Methods

### Plasma and cerebrospinal fluid samples

With the approval of the University of Colorado Institutional Review Board (COMIRB; No. 00-688, No. 13-3007), plasma and cerebrospinal fluid from MS and control patients with other central nervous system disorders were collected at the University of Colorado Hospital. Plasma was collected after centrifugation of blood samples at 2,000 × *g* for 10 min; CSF was immediately centrifuged at 500 × *g* for 10 min, and the supernatant was collected. Both CSF and plasma were stored at − 80°C until use. Samples were stored at − 80°C for 1–3 years before analysis. Previous studies indicate that long-term storage has a minimal impact on IgG glycosylation (11).

Patient demographics, including age and sex, are listed in **Table 1**, and **Table 2**.

**Table 1:** Patient demographics and details on samples used for experiments.

Patient ID	Diagnosis	Age	Sex	CSF cells	OCBs	Paired plasma	DMT	Assays
MS 7521	PPMS	62	M	ND	8	Yes	None	TEM, CDC, mass spec, IEF
MS 2243	PPMS	33	M	ND	17	Yes	None	TEM, CDC, mass spec, IEF
MS 5129	PPMS	55	M	ND	9	Yes	None	TEM, CDC, mass spec, IEF
MSR 0-1	RRMS	59	M	ND	ND	Yes	Rituximab	Protein aggregates, CDC, PNGase digestion, IEF
MSR 0-2	RRMS	34	F	ND	ND	Yes	Rituximab	Protein aggregates, CDC, PNGase digestion, IEF
MSR 0-3	RRMS	55	M	ND	ND	Yes	Rituximab	Protein aggregates, CDC, PNGase digestion, IEF
MSR 0-4	RRMS	55	F	ND	ND	Yes	Rituximab	Protein aggregates, CDC, PNGase digestion
MSR 0-5	RRMS	30	F	ND	ND	Yes	Rituximab	Protein aggregates, CDC, PNGase digestion
MSR 0-8	RRMS	29	F	ND	ND	Yes	Rituximab	Protein aggregates, CDC, PNGase digestion
MSR 0-9	RRMS	71	F	ND	ND	Yes	Rituximab	Protein aggregates, CDC, PNGase digestion
MSR 0-10	RRMS	39	M	ND	ND	Yes	Rituximab	Protein aggregates, CDC, PNGase digestion
MSR 0-11	RRMS	31	F	ND	ND	Yes	Rituximab	Protein aggregates, CDC, PNGase digestion
MS 02-14	RRMS	51	F	10	5	No	None	DeglycoMx digestion and IEF
MS 02-24	RRMS	39	F	17	1	No	None	PNGase and DeglycoMx digestions, IEF, Western blot
MS 04-03	RRMS	44	M	4	2	No	None	PNGase and DeglycoMx digestions, IEF
MS 04-05	RRMS	56	F	10	3	No	None	PNGase and DeglycoMx digestions, IEF
MS 05-01	RRMS	52	M	9	5	No	None	DeglycoMx digestion and IEF
MS 05-04	RPMS	57	F	6	22	No	None	DeglycoMx digestion and IEF
MS 07-01	RRMS	46	F	10	14	No	None	DeglycoMx digestion and IEF
OND 6124	Lupus	53	F	N/A	0		N/A	TEM, mass spec, CDC
OND 9180	Psoriasis	55	F	N/A	0		N/A	TEM, mass spec, CDC
NIC-1	Leukoencephalopahty	35	F	N/A	0		N/A	CDC
NIC-2	Cavernous hemangioma	61	F	N/A	0		N/A	CDC
NIC-3	Hypertension	60	F	N/A	0		N/A	CDC

*ND*, OCB data were not available from clinical records for these patients.

**Table 2:** Plasma samples for native Western blots.

Diagnosis	Age (mean)	Female (*n*)	Male (*n*)	Assay
MS	47.7	22	10	Native Western blots
HC	47.25	9	11	Native Western blots

### Characterization of IgG aggregates

#### Transmission electron microscopy

Transmission electron microscopy (TEM) was used to demonstrate the presence of IgG aggregates in three MS and three control CSF samples. The TEM imaging procedure was the same as previously described (21). Samples were prepared on continuous carbon films supported on 200-mesh copper grids (Ted Pella, Redding, CA). A 2.5-μl drop of MS or control CSF was applied to a freshly glow-discharged grid for 20 s and blotted with filter paper. The grid was washed once by dipping it in a water droplet for 15 s, then stained with a droplet of 2% (w/v) uranyl acetate for 1 min. Grids were blotted after each incubation and then air-dried to remove excess stain solution. Stained grids were imaged using a Thermo Fisher Talos L120C transmission electron microscope outfitted with a LaB6 filament and operating at an acceleration voltage of 120 kV. Automated data collection was performed using Leginon, with images recorded using a Ceta CMOS detector at a magnification of × 36,000 (3.98 Å/pixel).

### Measurements of protein aggregates

The ProteoStat Protein Aggregation Assay Kit (ENZ-51023-KP002, Enzo, Farmingdale, NY) was used to evaluate protein aggregate levels. CSF (5 µl) and paired plasma (1:300 dilution) were incubated with the assay buffer, followed by a measurement of the fluorescence intensity values at 550/600 with a fluorescence microplate reader.

### Evaluation of neuronal cytotoxicity induced by MS CSF and IgG aggregates

#### Human iPSC-derived neurons

Human induced pluripotent stem cells (iPSCs) were derived from a 27-year-old healthy man. First, the iPSCs were converted into neural precursor cells (NPCs). Next, the NPCs were cultured in neural induction medium (No. 05839, STEMCELL Technologies, Vancouver, BC, V6A 1B6, Canada). Finally, the cells were replated and maintained in neural maturation medium (No. 08605, STEMCELL Technologies). The immature neurons were plated at a density of 30 k/cm^2^ in a 96-well plate. The plates were coated with poly-l-ornithine (15 µg/ml) and laminin (5 µg/ml). Medium was changed every 2–3 days.

#### Neuroblastoma cell line SH-SY5Y

The SH-SY5Y neuroblastoma cells were obtained from the American Type Culture Collection (ATCC) (No. CRL-2266). Cells were cultured in DMEM with 10% fetal bovine serum (FBS) and 1× penicillin–streptomycin on 0.1% gelatin-coated plates and passaged using a 0.05% Trypsin and 0.02% EDTA solution. Cells at passage numbers 3–10 were used for cytotoxicity assays. For MS aggregate treatments, cells were plated at 20,000 cells/cm^2^ in 96-well plates.

### Evaluation of neuronal cytotoxicity induced by MS CSF and IgG aggregates

The RealTime-Glo Annexin V Apoptosis and Necrosis Assay Kit (No. JA1011, Promega, Madison, WI) was used to evaluate apoptotic and necrotic cell death. The five components (stock concentration at 1,000 ×) were diluted to 1 × in the culture medium and added to the treatment mixtures. Apoptosis was measured by luminescence, and necrosis by green fluorescence (excitation/emission: 485/528 nm).

Human iPSC-derived neurons and SH-SY5Y neuronal cells (No. CRL-2266, ATCC), at 30%–50% confluency, were used for treatment. The following components were mixed in a 96-well U-bottom sterile plate (No. 6018-P113, Greiner Bio-One, Bad Haller Str. 324550 Kremsmünster, Austria): (1) samples (plasma, CSF, or enriched IgG aggregates); (2) 5% normal human serum (NHS) as a source of complement (No. NHS, Complement Technology Inc., Tyler, Texas); (3) 1 × assay reagents (apoptosis/necrosis assay, live/dead assay, or propidium iodide); (4) culture medium to a final volume of 100 µl for 96-well plates. After removing the spent media, the cells were washed once with fresh, warm medium, followed by incubation with the treatment mixtures. Cytotoxicity was measured every 30–60 min for 4–24 h. All readings were done using a Biotek Synergy H4 hybrid reader (software version Gen5 3.09) (21).

### Treatments of samples for downstream applications

#### Collection of retentates and filtrates after 300 kDa filtration of CSF

We used a 300-kDa centrifugal device, Nanosep 300 K OMEGA (No. OD300C34, Pall Life Science, 25 Harbor Park Drive in Port Washington, New York), to collect the IgG aggregates in the retentates. MS and control CSF were centrifuged for 10 min at 6,000 × *g*, and both the filtrates and the retentates (in PBS) were collected and stored at − 80°C.

#### Disruption of IgG aggregates

MS CSF was incubated with 8 M urea (No. 51457, Sigma, Burlington, Massachusetts), 0.2 M glycine/HCl, pH 2.8, or PBS (1:3 sample-to-buffer ratio, v/v) at 37°C for 1 h. The treated CSF samples were either used directly in experiments or stored at − 80°C for future use.

#### Depletion of IgG1 in MS CSF

MS CSF was incubated with biotinylated mouse antihuman IgG1 antibody (No. B6775, Sigma, Carlsbad, California) at 4 mg/ml overnight at 4°C. M-280 Streptavidin-conjugated Dynabeads (No. 11205D, Invitrogen, Carlsbad, California) were used to capture the biotinylated antibodies (depletion). The unbound solutions were collected as IgG1-depleted.

#### Enzymatic deglycosylation

PNGase F is the most effective enzymatic method for removing almost all *N*-linked oligosaccharides from glycoproteins. We used PNGase F (New England Biolabs, 240 County Road, Ipswich, MA 01938, USA) to treat MS CSF. We used the Enzymatic Deglycosylation Kit (QA Bio, Palm Desert, CA 92260, US), a premixed cocktail of enzymes required to remove all N-linked oligosaccharides and most O-linked sugars from glycoproteins, to remove glucans from MS CSF proteins. We followed the manufacturer’s protocols for the enzymatic treatments.

#### Isoelectric focusing immunoblot for the detection of oligoclonal bands

We used similar procedures as described previously for the detection of oligoclonal bands (22). Neat CSF (5 µl) and paired plasma (1:300 dilution), or concentrated CSF, or treated CSF were used for isoelectric focusing (IEF) with the SPIFE^®^ IgG IEF Kit (Helena Laboratories, Beaumont, Texas) on a SPIFE 3000 electrophoresis analyzer. Wicks were soaked in an anode (0.3 M acetic acid) or cathode (1 M NaOH) solution and applied to the edge of a SPIFE^®^ IgG IEF gel. Five microliters of concentrated MS CSF/sera (3–5 μg IgG for phage probe and 100 ng IgG for alkaline phosphatase-conjugated antihuman IgG probe, Beaumont, Texas) were loaded into wells of a SPIFE IEF gel. After electrophoresis at 700 V for 1 h at 15°C, the samples were transferred to Polyvinylidene fluoride (PVDF) membranes for 45 min, followed by blocking in Helena blocking agent (1 g bovine milk protein/50 ml 1 × Tris-buffered saline (TBS)) for 1 h at room temperature. Membranes were incubated with the respective phage peptide at concentrations ranging from 5.0 × 10^10^ to 1.5 × 10^11^ pfu/ml in 1:10 Helena blocking agent/TBST (blocking buffer) at room temperature for 2 h. After washing with 0.05% Tween-TBS, membranes were incubated with mouse anti-M13 mAb (New England Biolabs) at a 1:500 dilution in blocking buffer. This was followed by a 1:500 dilution of AP-conjugated anti-mouse IgG at room temperature for 1 h. Membranes were developed with NBT/BCIP substrate (Roche, Basel, Switzerland) (22).

#### Mass spectrometry proteomic analysis of CSF

We used the same procedure as described previously for CSF proteomics (21). The raw mass spectrometry proteomics data have been deposited in the MassIVE repository (https://massive.ucsd.edu). The submission ID is MSV000100808.

### Determination of the levels of IgG glycosylation

#### IgG1 captured ELISA to detect galactosylation

ELISA plates (No. 437796, Thermo Scientific, Waltham, MA) were coated with 50 mg/ml mouse antihuman IgG1 mAb (No. I2513, Sigma) in 0.1 M sodium bicarbonate (No. 28382, Thermo Scientific) overnight at 4°C. Wells were blocked with either casein buffer (to detect IgG1; No. SP-5020, Vector Labs) or carbo-free buffer (to detect galactose; No. SP-5040, Vector Lab, 6737 Mowry Ave Newark, CA 94560). Paired CSF and plasma samples diluted (1:10 and 1:3,000, respectively) in TBS were added to the wells and incubated for at least 1 h at room temperature with gentle shaking. To detect IgG1 galactosylation, biotinylated *Ricinus communis* agglutinin I (RCA) (1 mg/ml in TBS; No. B-1085-1, Vector Labs) was used. IgG1 detection was achieved by incubation with goat antihuman IgG-Fc (1:1,000 in TBS; No. 609-1603, Rockland Immunochemicals, Inc. 6737 Mowry Ave Newark, CA 94560). NeutrAvidin-HRP (No. 31001, Thermo Scientific) was used as the secondary antibody at dilutions of 1:1,000 for IgG-Fc and 1:10,000 for RCA. The colorimetric reaction was performed using TMB (100 ml/well; No. 5120-0047, SeraCare: Milford, Massachusetts) and stopped with 0.1 N HCl. OD at 450 nm was measured using a BioTek Synergy 2 plate reader.

### Lectin Western blots

Mini-PROTEAN^®^ TGX 4%–12% Gels (BioRad, Hercules, California) were used for SDS-PAGE analysis with 1 × Tris/glycine/SDS running buffer (BioRad). Neat CSF and plasma (1:300 dilution) were denatured and reduced by incubation with 1 × lane marker reducing sample buffer containing dithiothreitol (Thermo Scientific) at 95°C for 10 min. Gels were electrophoresed for 40 min and blotted onto a PVDF membrane (BioRad). Membranes were blocked for 1 h with 1 × carbo-free blocking buffer (Vector Labs). Lectin binding was detected with 1 μg/ml biotinylated elderberry bark lectin *Sambucus nigra* (SNA) or 1 μg/ml biotinylated RCA for 1 h at room temperature. All lectin probes were from Vector Labs. Membranes were then incubated with Thermo Fisher High Sensitivity NeutrAvidin^®^-HRP for 1 h at room temperature at a dilution of 1:50,000 for both SNA and RCA blots, followed by SuperSignal^®^ West Pico substrate for chemiluminescent detection (17).

### Native gel Western blots

We used the Invitrogen NativePAGE Bis-Tris Gel System for plasma native gel Western blots. Lectin binding was detected with 2 μg/ml biotinylated SNA or 2 μg/ml biotinylated RCA overnight at 4°C. NeutrAvidin^®^-HRP (1:20,000) was used as a secondary antibody for 1 h at room temperature, followed by detection with SuperSignal^®^ Femto substrate.

### Statistical analysis

All experiments were repeated at least twice. Statistical analyses were performed using GraphPad Prism (version 10). A one-way analysis of variance (ANOVA) test, followed by a *post-hoc* Tukey’s multiple comparisons test, was performed to determine the significance of the results. The Mann–Whitney *U* test was used for unpaired samples. A Tukey-adjusted multivariate ANOVA (MANOVA) was used to simultaneously compare each of the four IgG subclasses to the control group. *p*-values < 0.05 were considered statistically significant, and all tests were two-sided.

#### Justification of sample size

We acknowledge the modest sample sizes, which reflect the inherent difficulty of obtaining MS CSF samples. The study was designed as a mechanistic investigation rather than a population-level analysis. Qualitative figures (e.g., TEM, IEF blots) are representative and intended to demonstrate structural and mechanistic differences, which do not require large cohorts. Importantly, all quantitative analyses—including aggregation assays, cytotoxicity tests, glycosylation ELISA, and proteomics—showed robust statistical significance (e.g., *p* < 0.0001 for aggregation assay; *p* < 0.001 for CSF cytotoxicity), indicating sufficient power to detect biologically relevant effects. Our sample sizes are comparable to those of previous MS CSF studies (9, 17), and the findings were reproducible across independent experiments.

## Results

### The CNS compartmentalized IgG aggregates in MS produce higher levels of complement-dependent neuronal cytotoxicity compared to paired plasma

**Figure 1:**
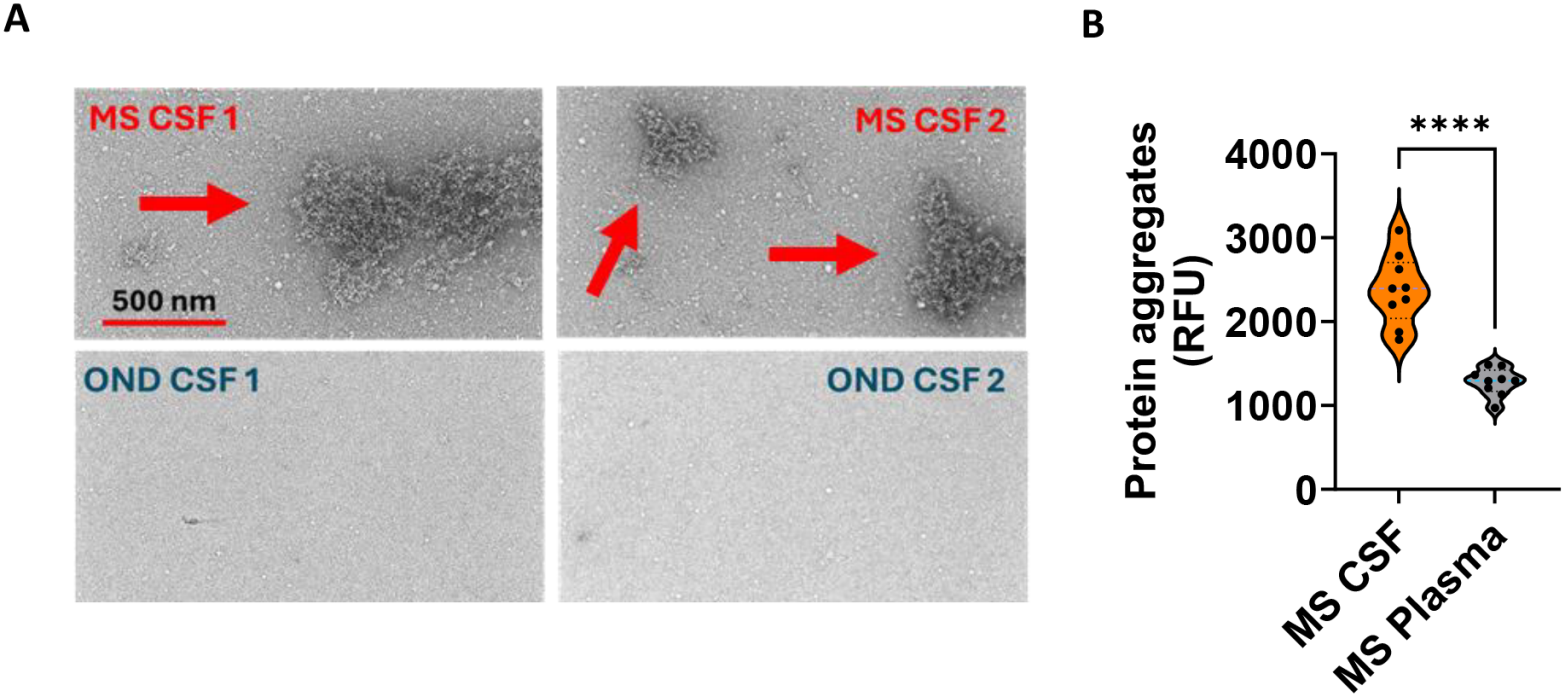
MS CSF contains higher levels of IgG aggregates compared to paired plasma. **(A)** Transmission electron microscopy images of IgG aggregates in PPMS CSF, but not in control CSF (OND1: lupus, OND2: psoriasis). Scale bar: 500 nm. **(B)** MS CSF contains significantly higher levels of protein aggregates compared to paired plasma. MS CSF (5 µl) and paired plasma (1:300 dilutions) (*n* = 9) were analyzed for the presence of protein aggregates using the Enzo Proteostat Protein Aggregation Assay Kit. The relative fluorescent unit (RFU, 550/600 nm) correlates with protein aggregate levels. Statistical test: Mann–Whitney *U* test, ^****^p < 0.0001.

We previously demonstrated that MS blood IgG aggregates induce complement-dependent cytotoxicity in primary neurons, primary astrocytes, neuroblastoma cell line SH-SY5Y, and newborn mouse brain slices (21). To determine the presence of IgG aggregates in MS CSF, we used TEM and the PROTEOSTAT^®^ Protein Aggregation Assay (Enzo). We demonstrated that MS CSF contains large aggregates (> 100 nm) compared to other neurological controls (**Figure 1A**) and significantly elevated levels of aggregates in MS CSF compared to paired plasma (*p* < 0.0001, *n* = 9) (**Figure 1B**).

**Figure 2:**
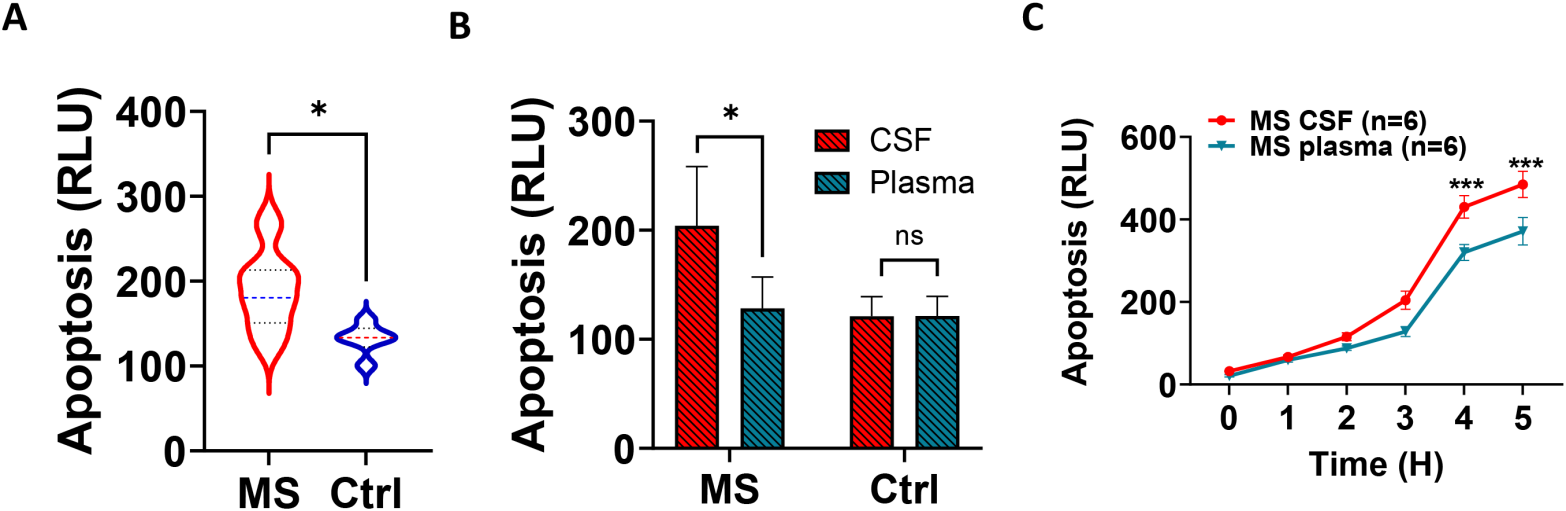
MS CSF induces greater complement-dependent neuronal apoptosis than paired plasma. **(A)** Apoptosis in iPSC-derived neurons treated with MS CSF vs. control CSF (*n* = 13 vs. *n* = 6), measured after 4 h using RealTime-Glo Annexin V assay. **(B)** MS CSF induced higher levels of cytotoxicity in neurons compared to paired plasma (4-h treatment). CSF (neat) and paired plasma (1:300 dilution) were used to treat neurons in the presence of NHS. MS: *n* = 6; Ctrl: *n* = 3. **(C)** Time-course graph showing significantly elevated levels of neuronal apoptosis in CSF compared with paired plasma (*n* = 6).

Furthermore, we showed that MS CSF induced higher levels of neuronal apoptosis than control CSF in other CNS disorders (**Figure 2A**). To demonstrate higher levels of MS CNS-compartmentalized neuronal cytotoxicity, we treated both the neuroblastoma cell line and IPS-derived neurons with neat CSF and paired plasma (1:300 dilution). We showed that MS CSF significantly increased neuronal apoptosis in both types of neurons compared with paired plasma (**Figures 2B, C**).

**Figure 3:**
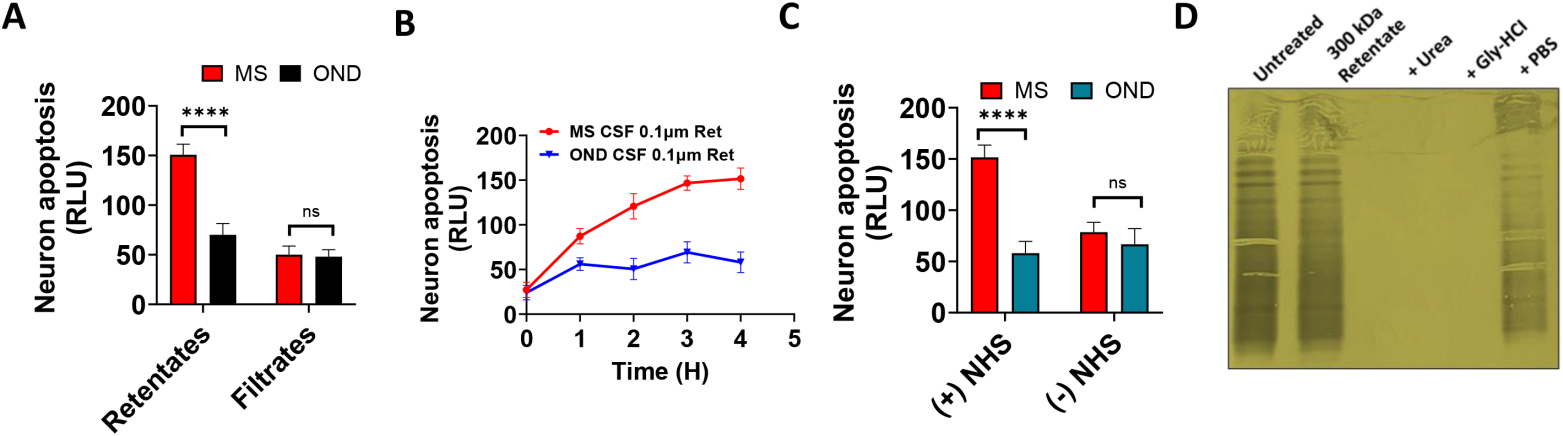
IgG aggregates (> 0.1 μm) in MS CSF drive complement-dependent neuronal cytotoxicity. **(A)** MS CSF IgG aggregates, not filtrates, induce neuronal apoptosis. MS CSF IgG aggregates were enriched by collecting the retentates after filtration with 100 nm columns. The neuronal cell line SH-SY5Y was treated with MS CSF aggregates and filtrates. **(B)** The time-course graph showed significantly higher levels of neuronal apoptosis induced by MS CSF IgG aggregates than by aggregates collected from OND CSF. **(C)** Normal human serum (NHS) is required for MS CSF IgG aggregate-induced neuronal cytotoxicity. SH-SY5Y cells were treated with MS and control CSF for 4 h in the presence or absence of NHS. Higher levels of neuronal apoptosis were observed in samples with NHS only. No differences in cytotoxicity were observed in samples treated without NHS (−NHS). **(D)** IEF blots show OCB retention in aggregates and loss after treatment with aggregate disruptors (8 M urea, 0.1 M Gly-HCl).

To demonstrate that large IgG aggregates contribute to neuronal apoptosis caused by MS CSF, we enriched MS CSF IgG aggregates by collecting the retentates after CSF filtration using 100-nm columns. We showed that MS retentates, not filtrates, induced neuronal apoptosis in SH-SY5Y cells (**Figures 3A, B**). These data support the notion that MS CSF IgG aggregates induce neuronal cytotoxicity. Consistent with blood IgG aggregates, we showed that NHS is required for MS CSF IgG aggregate-induced neuronal cytotoxicity (**Figure 3C**). We previously showed that MS blood IgG aggregates treated with 8 M urea or 0.1 M Gly-HCl lost neuronal cytotoxicity (11). To demonstrate that treatments of MS CSF with aggregate disruptors may result in the loss of oligoclonal bands, we treated MS CSF, followed by an IEF blot probed with antihuman IgG (H + L). We demonstrate that OCBs are retained in aggregates, and treatment with aggregate disruptors leads to the loss of OCBs (**Figure 3D**).

### Immunoglobulin G, immunoglobulin A, and complement activation products are the major proteins in the MS CNS compartment

**Figure 4:**
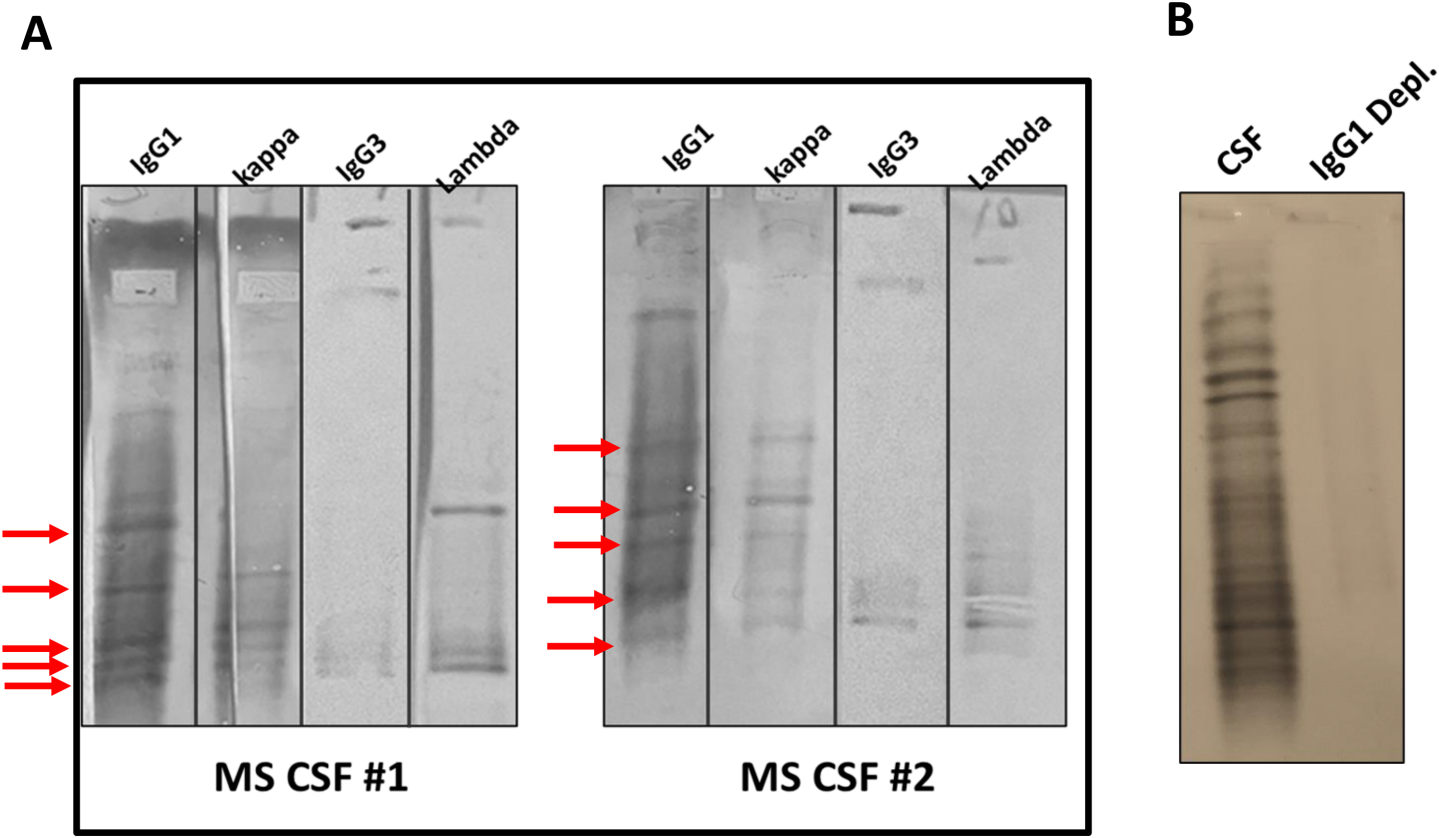
IgG1 is the dominant component of OCBs in MS CSF and coexists with IgG3, kappa, and lambda chains. **(A)** MS CSF was focused by IEF, followed by immunoblotting with anti-IgG1, anti-IgG3, anti-kappa, and anti-lambda antibodies, followed by incubation with HRP-secondary antibodies and color detection. Overlapping OCB bands were observed in IgG1, IgG3, kappa, and lambda chains (arrows). Two representative MS CSF blots are shown. **(B)** IgG1 depletion abolishes all OCBs, confirming its major role. MS CSF was incubated with biotinylated antihuman IgG1 antibody, followed by depletion with M-280 streptavidin-conjugated Dynabeads to remove all IgG1 antibodies. The IgG1-depleted MS CSF IEF blot was probed with anti-IgG (H + L). The original (neat) CSF serves as a control.

The presence of elevated levels of aggregates in MS CSF compared to the paired plasma suggested that they may contribute to oligoclonal bands. We performed IEF blots of CSF and paired plasma (1:300 dilution), probed with anti-IgG1, anti-IgG3, anti-kappa, and anti-lambda antibodies. We showed the co-presence of IgG1, IgG3, kappa, and lambda light chains in the same OCB bands, suggesting that each OCB may contain multiple IgG subclasses (**Figure 4A**), which is consistent with a previous report (23). We further showed that depletion of IgG1 from MS CSF resulted in loss of OCBs (**Figure 4B**), supporting previous studies showing that IgG1 is the major component of OCBs in MS (23).

**Figure 5:**
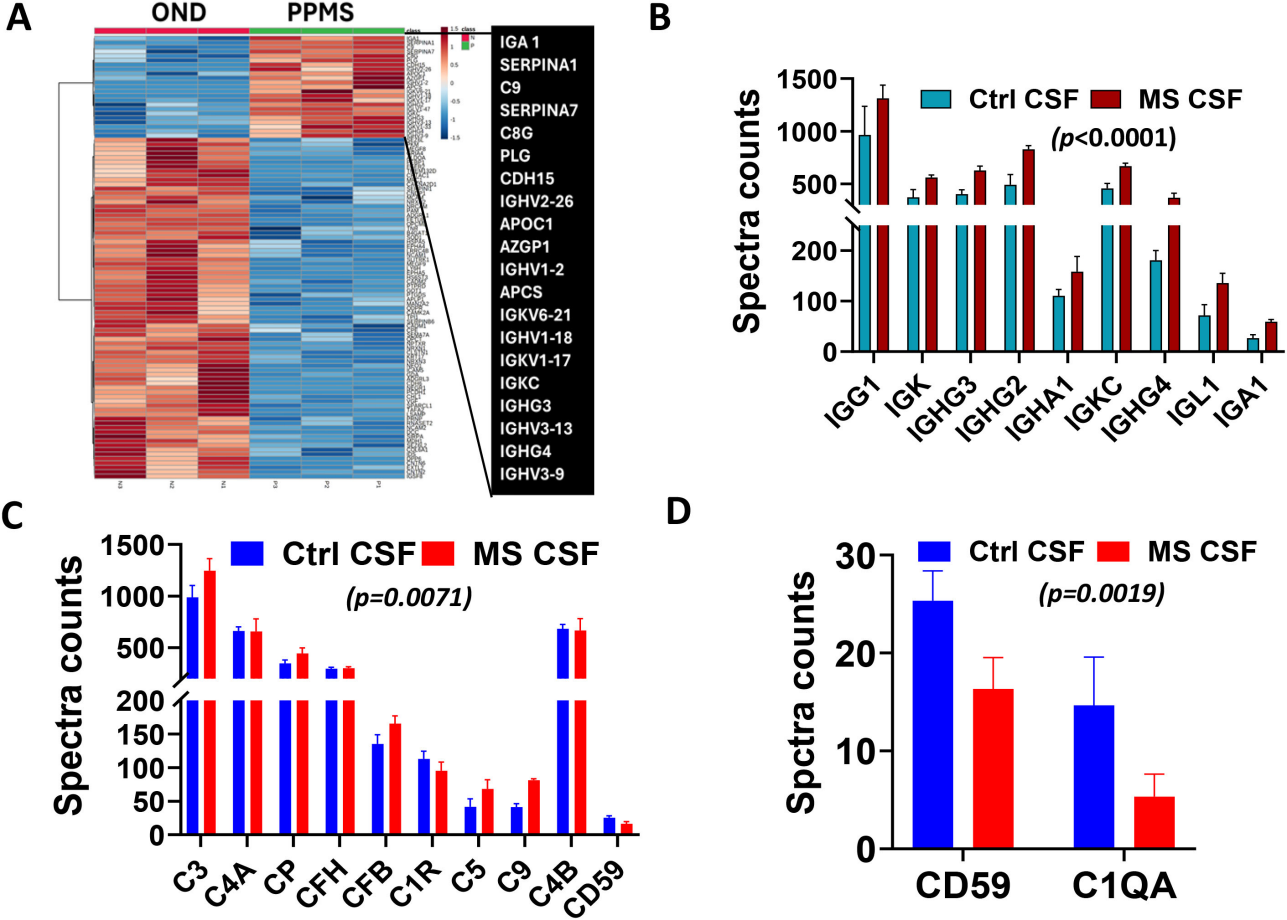
Proteomic analysis reveals enrichment of IgG subclasses and complement activation in MS CSF. **(A)** The heatmap shows IgG subclasses and complement enrichment in MS CSF. PPMS = 3, OND = 3 (systemic lupus erythematosus, psoriasis, and leukoencephalopathy). **(B)** Proteomics spectra count comparison of immunoglobulins between MS and control CSF. Significantly higher levels of IgG were present in MS CSF compared to controls (two-way ANOVA, *p* < 0.0001). **(C)** Spectra count comparison of the complement between MS and controls. MS CSF had higher levels of complement activation compared to controls (two-way ANOVA, *p* = 0.0071). **(D)** The protectin (CD59) and the complement component C1q were lower in MS CSF than in controls (*p* = 0.0019).

We further carried out mass spectrometry proteomics on three primary progressive (PPMS) CSF samples and three controls with other neurological disorders (OND) CSF samples. Compared to controls with other neurological diseases, MS CSF contained significantly higher levels of IgG heavy and light chains and complement (**Figure 5A**). Analysis of spectra counts showed a highly significant elevation of immunoglobulins in MS CSF (**Figures 5B**, ANOVA *p* < 0.0001). For complement activation, we found significantly higher levels of complement activation components in MS CSF compared to controls (**Figures 5C**, two-way ANOVA, *p* = 0.0071). Interestingly, CD59 (protectin) and C1qA were lower in MS CSF than in controls (**Figures 5D**, p = 0.0019).

### Highly glycosylated IgG is present in the CNS compartment of MS and other neurological disorders

**Figure 6:**
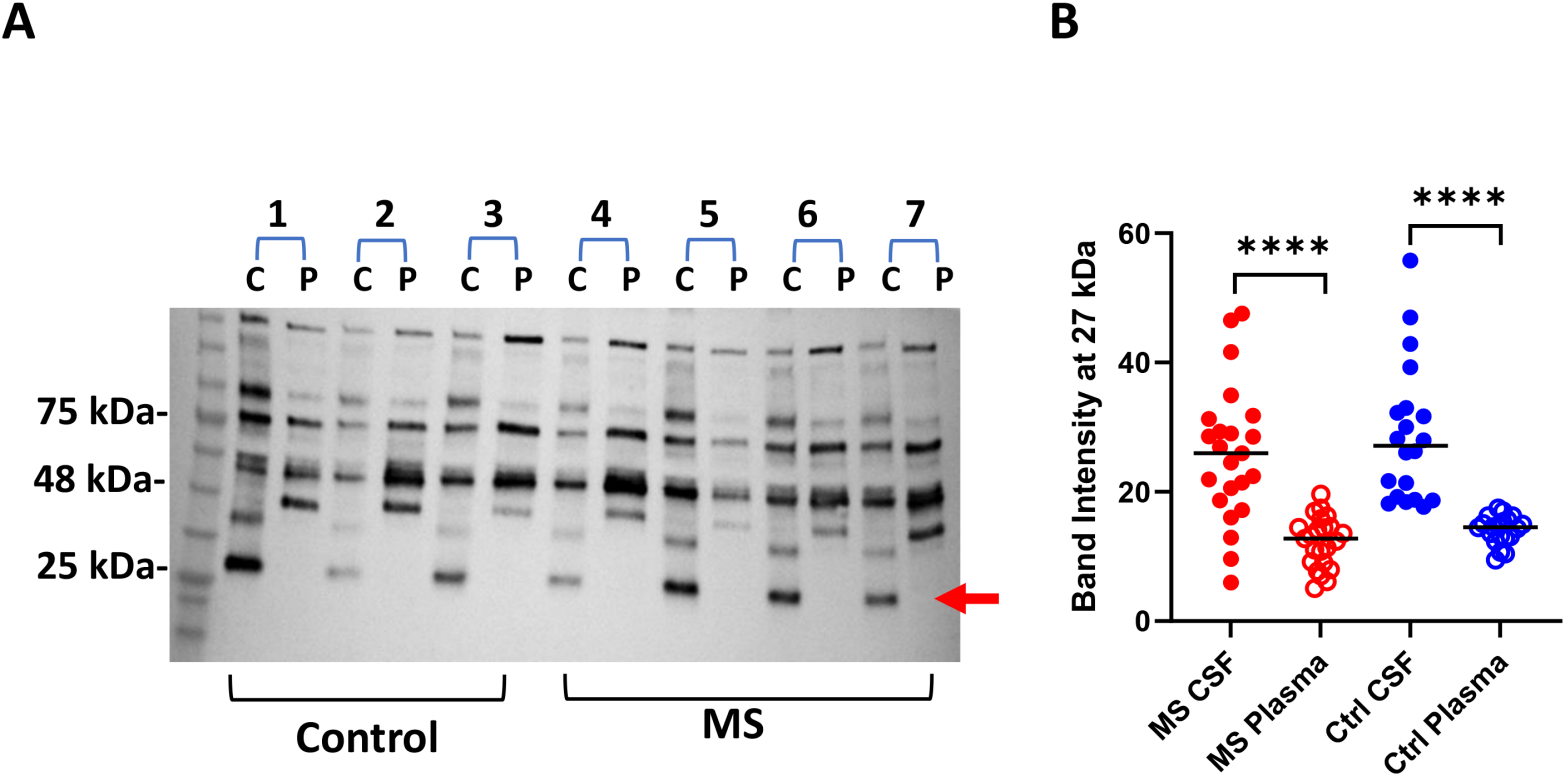
Lectin blot detecting galactose (RCA) and sialic acid (SNA). **(A)** SDS-PAGE Western blots demonstrate highly elevated glycosylated IgG in the CNS compartment compared to paired plasma. A representative Western blot image shows that the galactosylated IgG light chain band (arrow, 25 kDa) was detected only in CSF, not in paired plasma, in both MS and control subjects. **(B)** Quantification of band intensity from 23 MS pairs and 20 OND pairs (^****^p < 0.0001). C, CSF; P, plasma.

We previously showed that significantly higher levels of sialylated and galactosylated IgG were present in MS plasma (21). To further determine the levels of glycosylated IgG in MS CSF and paired plasma, we performed an IgG1 capture ELISA to evaluate the levels of galactosylated IgG using biotinylated RCA. MS CSF contained significantly higher levels of galactosylated IgG1 compared to paired plasma (**Figures 6A**, p = 0.0012). We also found higher levels of galactosylated IgG1 in control CSF (**Figures 6A**, p = 0.0015). However, we did not detect a significant difference in IgG1 antibodies when detected with anti-Fc antibody (data not shown). We confirmed higher levels of IgG glycosylation in MS CSF using Western blots with paired CSF and plasma samples. In both MS and controls, CSF showed significantly elevated galactose levels in IgG heavy chains (100, 75, and 55 kDa) and light chain 25 kDa bands (**Figure 6B**).

**Figure 7:**
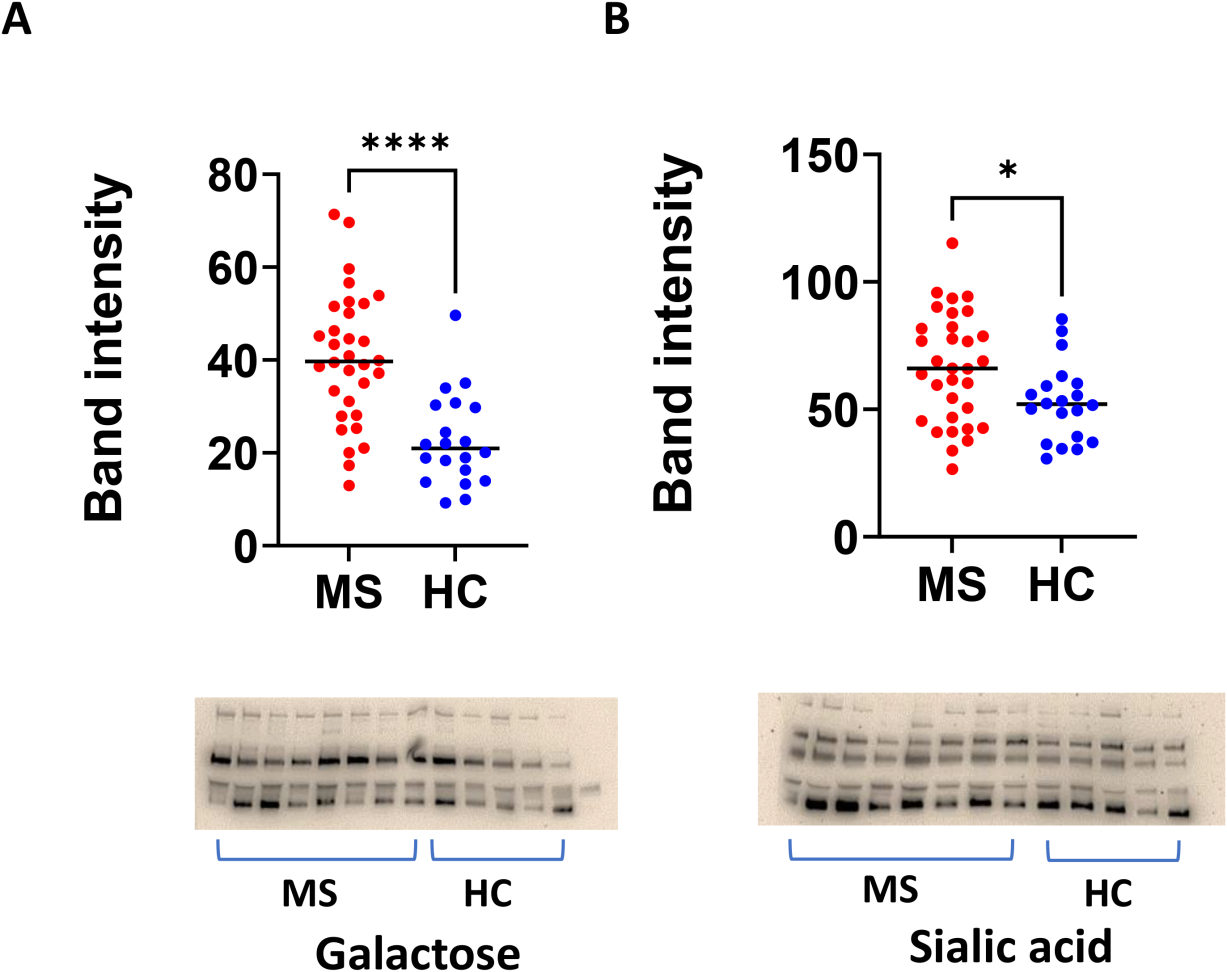
Native Western blots reveal increased galactosylated and sialylated IgG in MS plasma compared to healthy controls. **(A)** MS and healthy control plasma were separated in native PAGE, followed by blotting and lectin probing for galactose. A representative image is shown below the summary. **(B)** Plasma native Western blot showing sialic acid levels in MS and controls. HC, healthy control.

Furthermore, we showed higher levels of galactosylated (*p* < 0.0001) and sialylated IgG in MS plasma compared to controls (**Figure 7**).

### Glycosylated IgG in MS CSF contributes to oligoclonal bands and induces neuronal apoptosis

**Figure 8:**
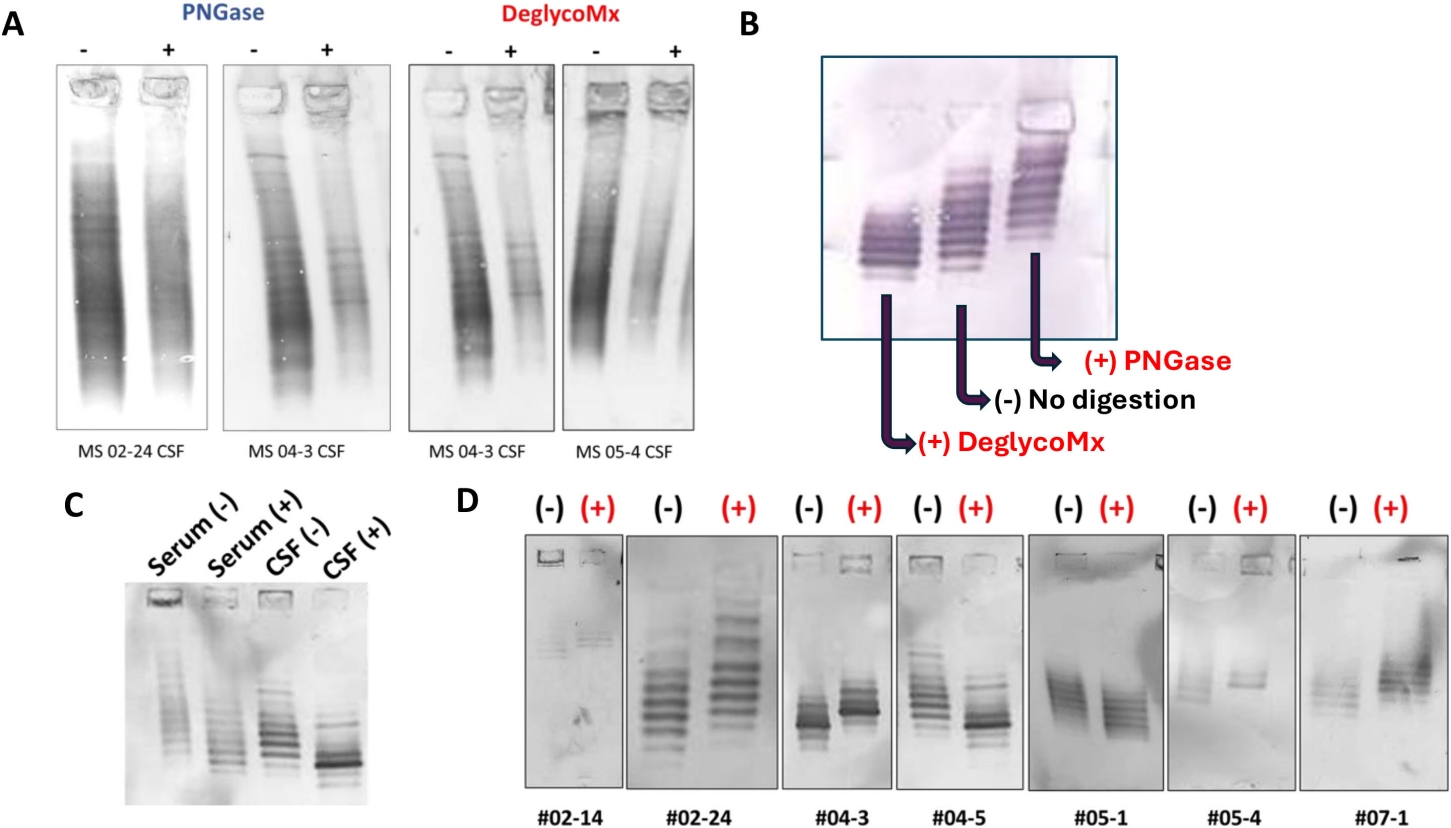
Removal of glycans reduces oligoclonal bands in MS CSF. MS CSF or plasma was digested with PNGase F or DeGlycoMx, followed by IEF blots probed with antihuman IgG or phage peptides. **(A)** MS CSF digested with PNGase F or DeGlycoMx shows a reduction of OCBs. Two representative MS were shown. “+” enzyme treatment. Control (−) indicates that no enzyme was included. **(B)** MS CSF digested with DeGlycoMx or PNGase showed reduced OCBs as detected by specific phage peptides (7). **(C)** IEF blot of representative MS CSF and plasma (MS 04-5), digested with DeGlycoMx, shows the reduction of OCBs in MS CSF compared to paired plasma. Phage peptide F12 was used for OCB detection (22). **(D)** Digestion with DeGlycoMx in multiple MS CSF shows the change of OCB (*n* = 7).

To determine if glycosylation contributes to MS CSF oligoclonal bands, we incubated paired MS CSF and plasma with PNGase F or DeGlycoMx to remove glycans. MS CSF digested with PNGase F or DeGlycoMx showed a reduced number of OCBs (**Figure 8A**). We utilized previously identified MS CSF IgG-specific phage peptides to evaluate the change in OCB patterns after glycan removal (22). MS CSF digested with DeGlycoMx or PNGase showed a reduced number of OCB as detected by specific phage peptides (**Figure 8B**). In addition, MS CSF lost more OCB bands compared to paired serum after digestion with DeGlycoMx (**Figure 8C**). The reduction and change of OCB patterns after digestion with DeGlycoMx were observed with every pair of CSF and plasma examined (**Figure 8D**). Nonetheless, we did not observe a correlation between OCB number and IgG aggregate-induced cytotoxicity or the levels of glycosylation, indicating that aggregate formation is independent of total OCB count.

**Figure 9:**
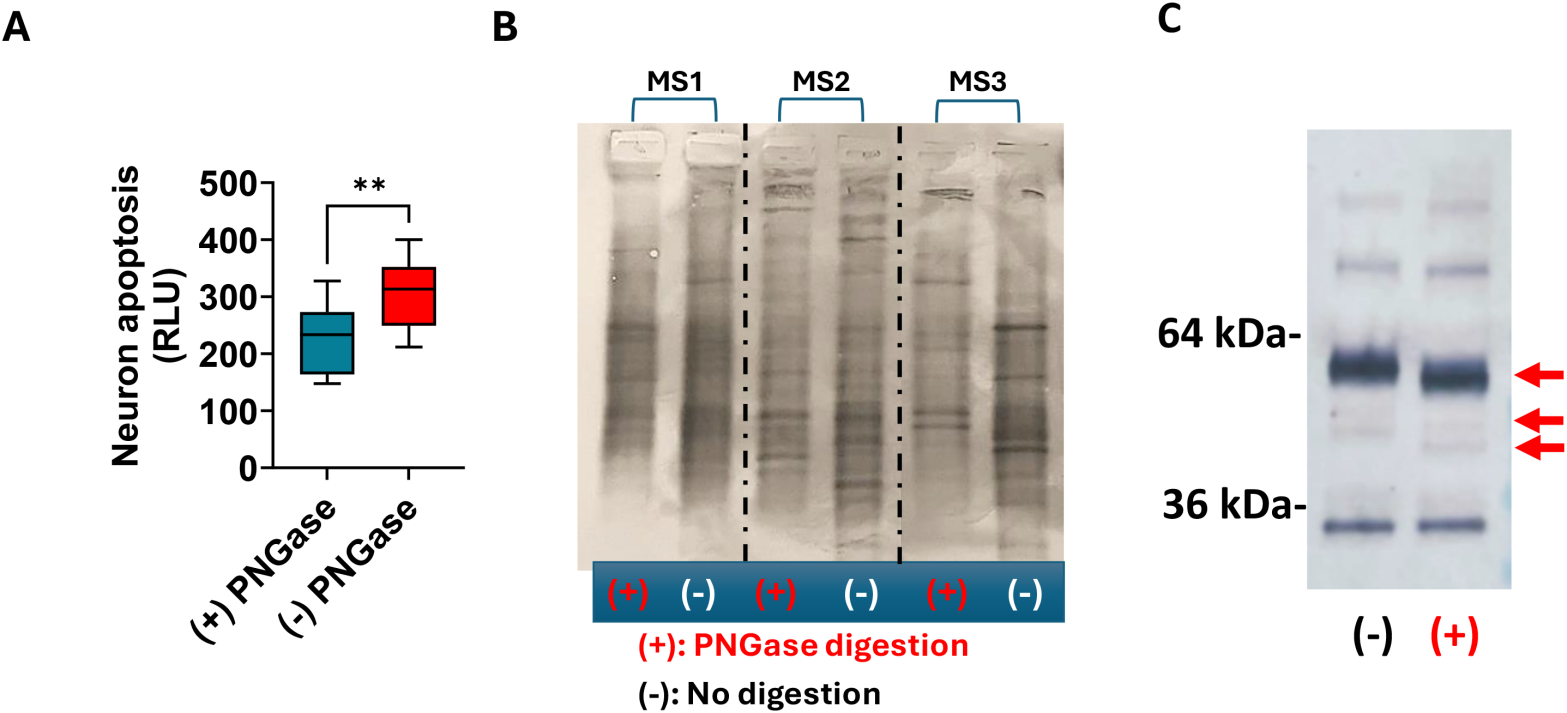
IgG glycosylation contributes to IgG aggregate-induced neuronal cytotoxicity and oligoclonal bands. MS CSF (*n* = 9) was incubated with PNGase F to remove N-linked oligosaccharide chains from IgG, followed by evaluation of neuronal cytotoxicity and oligoclonal bands. **(A)** Deglycosylation with PNGase F significantly reduces MS CSF-induced neuronal apoptosis. **(B)** IEF blot shows reduced OCBs after PNGase F treatment. **(C)** Western blot of deglycosylated MS CSF shows the reduction in the IgG heavy chain size. A representative MS CSF (MS 02-24) was incubated with PNGase F, followed by a Western blot to detect IgG (H + L).

We previously showed that MS IgG aggregates in the blood induce complement-dependent neuronal apoptosis (21). To determine whether IgG glycosylation contributes to neuronal cytotoxicity, we treated MS CSF with PNGase F to remove N-linked oligosaccharides, then assessed neuronal apoptosis. We showed that removing IgG glycans significantly reduced neuronal cytotoxicity (**Figure 9A**). The change of OCBs after digestion is shown in **Figure 9B**. The removal of glycans in IgG is demonstrated in Western blots probed with anti-IgG (H + L) (**Figure 9C**).

## Discussion

Intrathecal IgG, intrathecal IgG synthesis, and oligoclonal bands are the most characteristic features of MS (24). Using multiple immune assays, we demonstrated the presence of highly elevated levels of IgG aggregates in MS CSF, which induce neuronal cytotoxicity in both induced pluripotent stem cell (iPSC)-derived neurons and the neuronal cell line SH-SY5Y. Our IEF data show that MS OCBs contain multiple IgG species, and mass spectrometry data demonstrate that MS CSF is enriched with immunoglobulins and complements, supporting the link between IgG aggregates and OCBs in MS. Furthermore, we showed that the elevated levels of MS CSF IgG glycosylation contribute to oligoclonal bands and complement-dependent neuronal cytotoxicity.

The presence of protein aggregates in MS CSF was demonstrated by transmission electron microscopy (**Figure 1**). Large protein aggregates (> 100 nm) were observed only in MS CSF, not in controls. Protein aggregation assay confirmed higher aggregate levels in MS CSF than paired plasma. We demonstrated that MS CSF IgG aggregates, not the filtrates, cause complement-dependent neuronal apoptosis (**Figure 3**). Other recent studies have shown that CSF IgG in PPMS is pathogenic, leading to motor disability and spinal cord pathology, including demyelination, impaired remyelination, reactive astrogliosis, and axonal damage (4). Our data support that MS CSF IgG in aggregate form is toxic to neurons, consistent with our previous report that MS blood IgG aggregates induce neuron cytotoxicity (21).

Protein aggregation and inclusion body formation have been shown in other neurodegenerative diseases, such as Alzheimer’s disease, Parkinson’s disease, Huntington’s disease, amyotrophic lateral sclerosis, and prion diseases (25, 26). Several studies have suggested a heterogeneous composition of OCBs and IgG immune complexes in the MS brain. IgG1 and IgG3 subclasses are found in the same band, indicating a microheterogeneous composition of the OCBs (27). We previously reported that no correlation exists between CSF IgG concentrations and the number of OCBs (28). This lack of correlation could be due to the small sample size (*n* = 91 for MS and *n* = 24 for controls) or the presence of CSF IgG in the form of aggregates or immune complexes contributing to OCBs. In addition, significantly higher amounts of bound IgG were eluted from the MS brain using high- or low-pH buffers (29). The antibody–antigen immunocomplexes were detected in foamy macrophages in the active lesion areas (30). Mehta et al. (31) reported the presence of IgG immune complexes in MS brain tissue from both white and grey matter. In the current study, IgG1, IgG3, kappa, and lambda were detected within the same OCBs, providing additional evidence that each OCB may contain multiple Ig species. Several RRMS patients were on rituximab (**Table 1**). Although B-cell depletion may reduce IgG synthesis, the findings of IgG aggregates and glycosylation were consistent across treated and untreated patients, suggesting that these features are intrinsic to MS pathology.

A previous study reported that mass spectrometry of protein A-purified IgG from MS showed lower levels of glycosylation in CSF compared to paired plasma in both MS and control patients (9). We confirmed this finding in our recent report, showing that protein A-purified CSF IgG in MS had significantly lower levels of glycosylation (sialylation) compared to paired plasma (17). Our current analysis of neat, unpurified CSF revealed significantly elevated glycosylation in MS CSF. Furthermore, we recently discovered that the IgG aggregates in MS were enriched after binding to protein A (21), rather than the IgG purified by protein A. Our approach using neat CSF preserves the native heterogeneity of IgG, including aggregates and immune complexes that are enriched in glycosylated forms. These aggregates may carry multiple glycosylated IgG molecules, amplifying lectin-based detection signals. Furthermore, glycosylated IgG aggregates may interact differently with protein A than monomeric IgG, leading to their exclusion during purification.

It was shown recently that protein A inhibits complement activation by interfering with IgG hexamer formation (32). The enrichment of MS IgG aggregates after binding to protein A suggests that, in MS, IgG1 and IgG3 antibodies may possess unique Fc regions and form aggregates or IgG complexes that prevent binding to protein A (21). We further demonstrated that the IgG antibodies in the A-FT (collected after contacting with protein A) caused complement-dependent neuronal cytotoxicity and can serve as blood biomarkers for MS and progressive MS (21, 33). These apparently contradictory studies reveal novel interactions between MS IgG molecules and protein A and highlight the unique characteristics of MS IgG antibodies (20).

While protein A’s primary binding site is on the Fc region, the presence and type of glycans at the Asn297 site can affect the strength and specificity of binding (31). In the current study, we evaluated the glycosylation patterns of MS IgG using neat, unpurified CSF and plasma. Western blot analysis revealed significantly elevated levels of IgG glycosylation in MS CSF compared to paired plasma. In both MS and controls, CSF showed significantly higher galactose levels in IgG heavy chains (100, 75, and 55 kDa) and light chain 25 kDa bands (**Figure 6B**). Furthermore, higher levels of galactosylated (*p* < 0.0001) and sialylated IgG were observed in MS plasma compared to controls (**Figure 7**). Significantly, the removal of glycans in MS CSF reduced the number of oligoclonal bands (**Figure 8**). Paired MS CSF and plasma were treated with PNGase F or DeGlycoMx, followed by detection of OCBs using IEF blots probed with antihuman IgG or phage peptides. These results provide evidence that higher levels of intrathecal IgG glycosylation contribute to OCBs, indicating that OCBs in MS may contain highly glycosylated IgG aggregates or IgG immune complexes (32). We speculate that deglycosylation likely alters IgG conformation and isoelectric point, disrupting aggregate stability and OCB formation.

Several lines of evidence suggest that IgG galactosylation influences the inflammatory potential of IgG by modulating binding affinities for downstream effector functions, such as complement activation and Fcγ receptors (11). IgG Fc galactosylation impacts classical complement pathway activation by promoting hexamerization of IgG, enhancing C1q, and increasing CDC (15). Galactosylated IgG1 immune complexes were suggested to inhibit C5a-dependent neutrophil activation (34). To determine whether IgG glycosylation contributes to neuronal cytotoxicity, we treated MS CSF with PNGase F to remove N-linked oligosaccharides, then assessed neuronal apoptosis. We showed that removing IgG glycans significantly reduced neuronal cytotoxicity (**Figure 9A**). The change in OCBs after digestion is shown in **Figure 9B**. The removal of glycans in IgG is demonstrated in Western blots probed with anti-IgG (H + L) (**Figure 9C**).

Our findings provide a mechanistic link between IgG aggregates, glycosylation, and MS pathology. IgG aggregates in MS CSF likely arise from intermolecular interactions facilitated by aberrant glycosylation, which stabilizes multimeric structures and promotes OCB formation. These aggregates exhibit enhanced binding to C1q, initiating the classical complement pathway and leading to deposition of C3b and formation of the membrane attack complex (C5b-9), which we demonstrated to cause complement-dependent neuronal apoptosis (21). Glycosylation further amplifies these effects by modulating IgG Fc conformation and effector functions. Specifically, galactosylation and sialylation patterns observed in MS CSF increase IgG hexamerization, thereby enhancing C1q binding and complement activation (14, 15). In addition to complement-mediated injury, glycosylated IgG aggregates may engage Fcγ receptors on microglia and macrophages, triggering phagocytosis, cytokine release, and oxidative stress that contribute to compartmentalized inflammation and cortical demyelination (3, 9). These mechanisms explain why OCBs are not merely markers of intrathecal IgG synthesis but represent pathogenic immune complexes driving neurodegeneration. Importantly, the persistence of aggregates and glycosylation-driven effector functions may underlie the limited efficacy of B-cell depletion therapies in progressive MS, highlighting the need for strategies targeting IgG glycosylation or aggregate disruption (10, 17). Collectively, our data support a model in which IgG aggregates and glycosylation synergistically promote complement activation and Fc receptor engagement, amplifying neuroinflammation and neuronal injury in MS.

## Conclusions

Our data provide novel evidence linking OCBs to IgG aggregates and the contribution of IgG glycosylation to MS pathophysiology, offering new opportunities for therapeutic targeting. The limitations include a modest sample size, a lack of longitudinal data, and an absence of *in vivo* validation.

## Data Availability

The datasets generated and/or analyzed during the current study are available from the corresponding author on reasonable request. The mass spectrometry proteomics data supporting this study are deposited in the MassIVE repository under the accession ID MSV000100808. The dataset is accessible at: https://massive.ucsd.edu.
